# Supplementary material for: Spatial layout characteristics of Northern Wei Luoyang: A spatial humanities analysis of the “Record of the Monasteries of Luoyang”
Source: PLoS One. 2025 May 9;20(5):e0320159. doi: 10.1371/journal.pone.0320159 (PMC12064038; doi:10.1371/journal.pone.0320159)
Supplement: S1 File — (PDF) [file pone.0320159.s001.pdf]

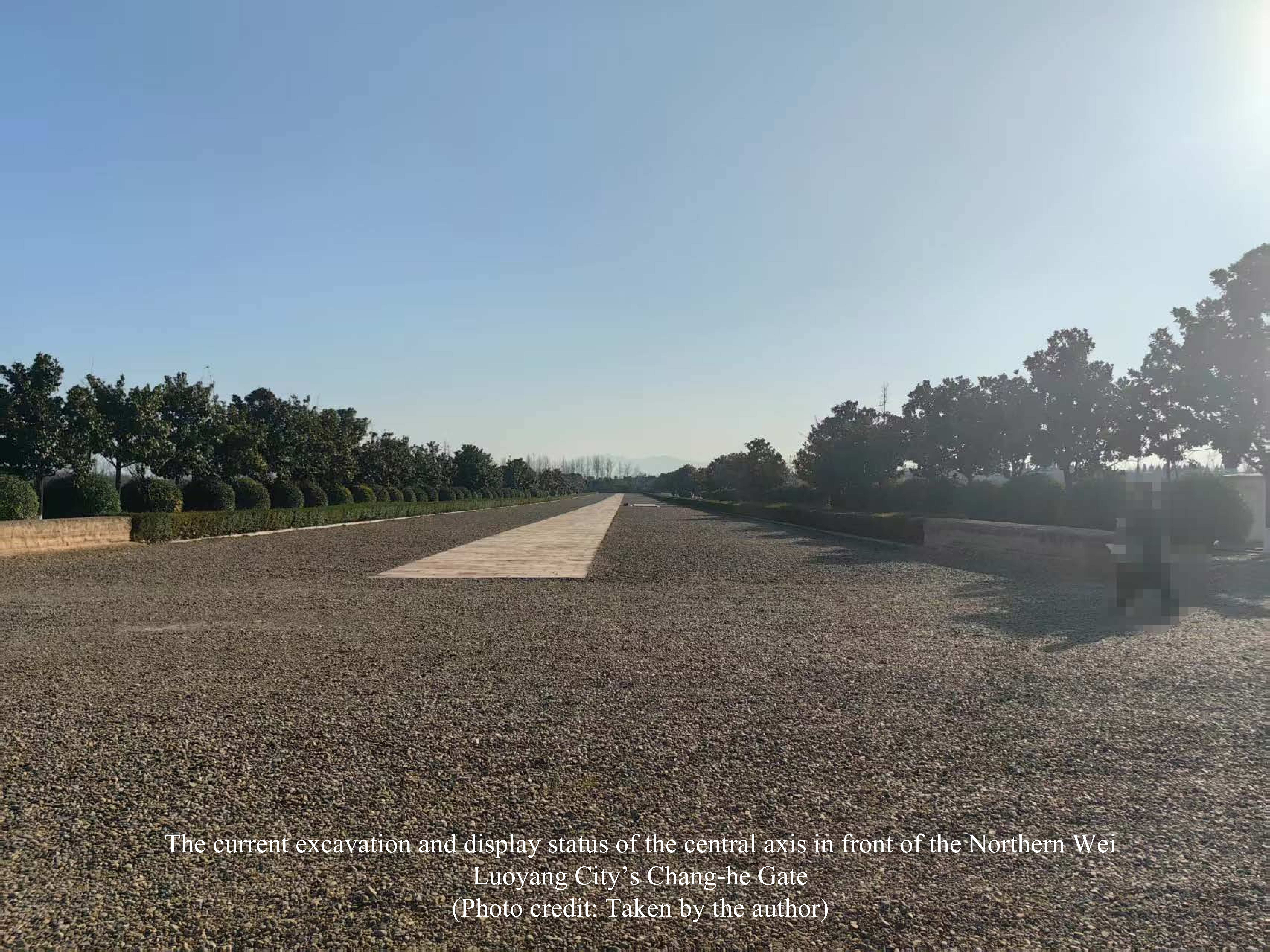

The current excavation and display status of the central axis in front of the Northern Wei Luoyang City's Chang-he Gate
(Photo credit: Taken by the author)

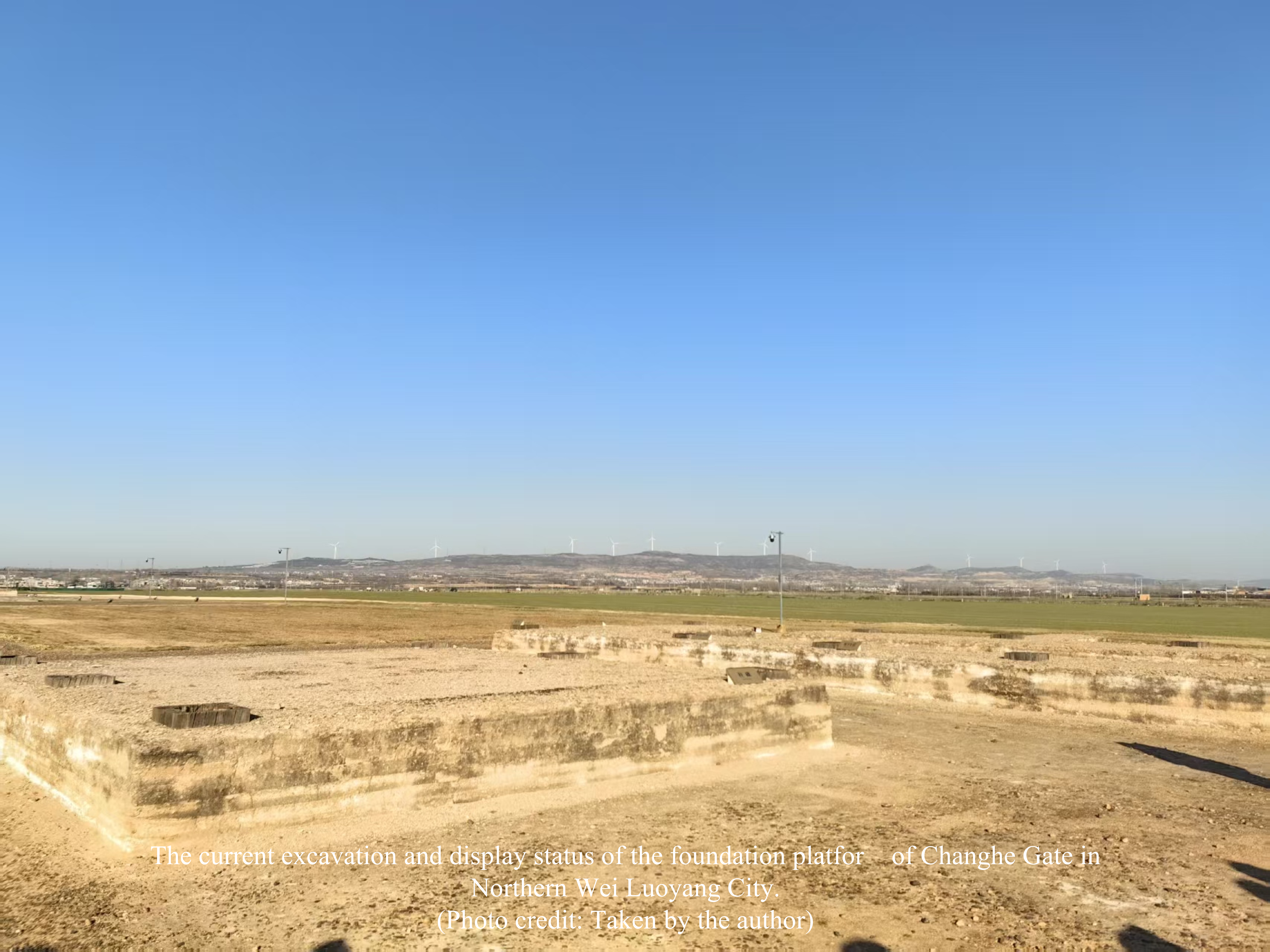

The current excavation and display status of the foundation platfor of Changhe Gate in Northern Wei Luoyang City.
(Photo credit: Taken by the author)

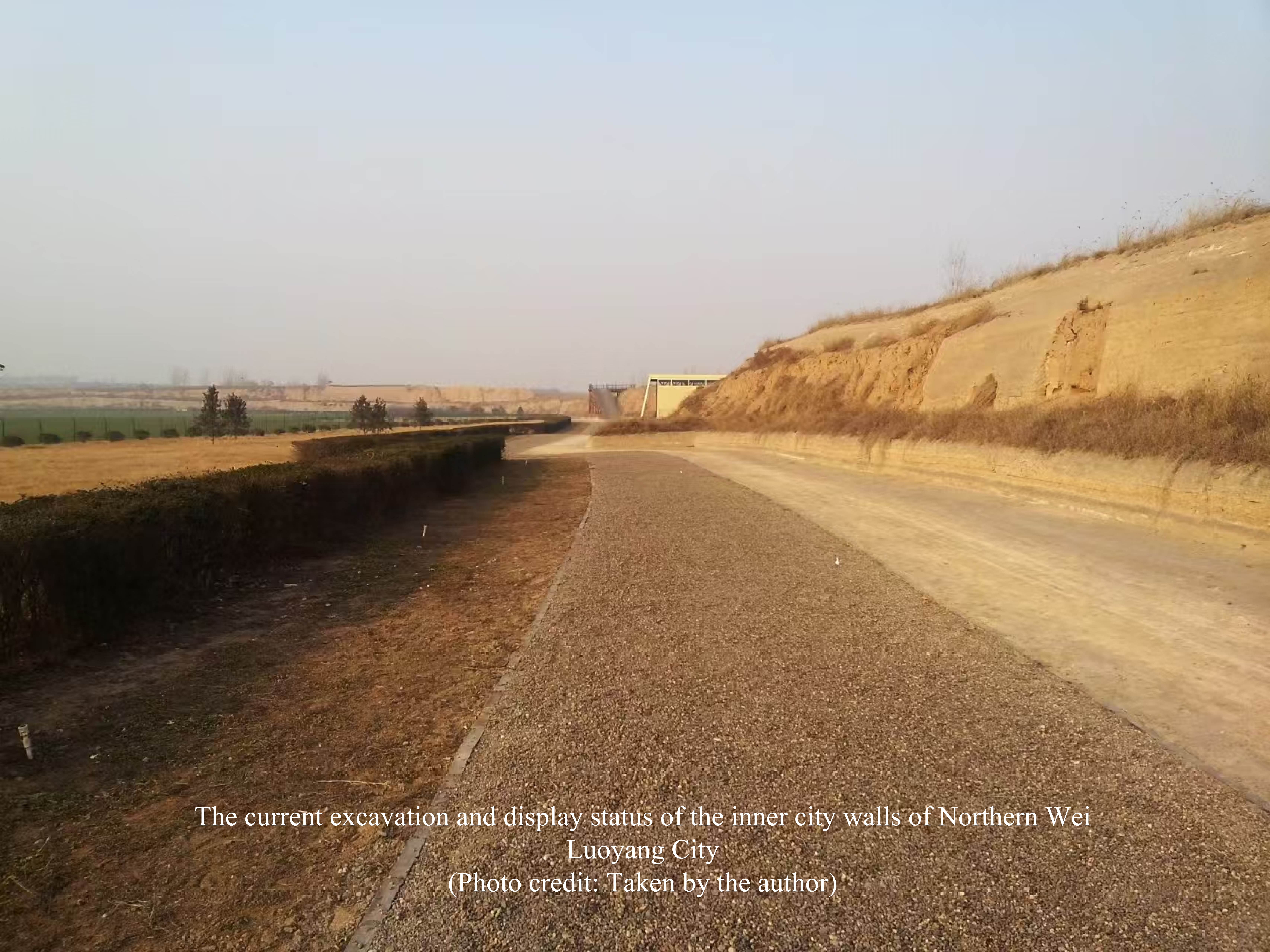

The current excavation and display status of the inner city walls of Northern Wei Luoyang City
(Photo credit: Taken by the author)

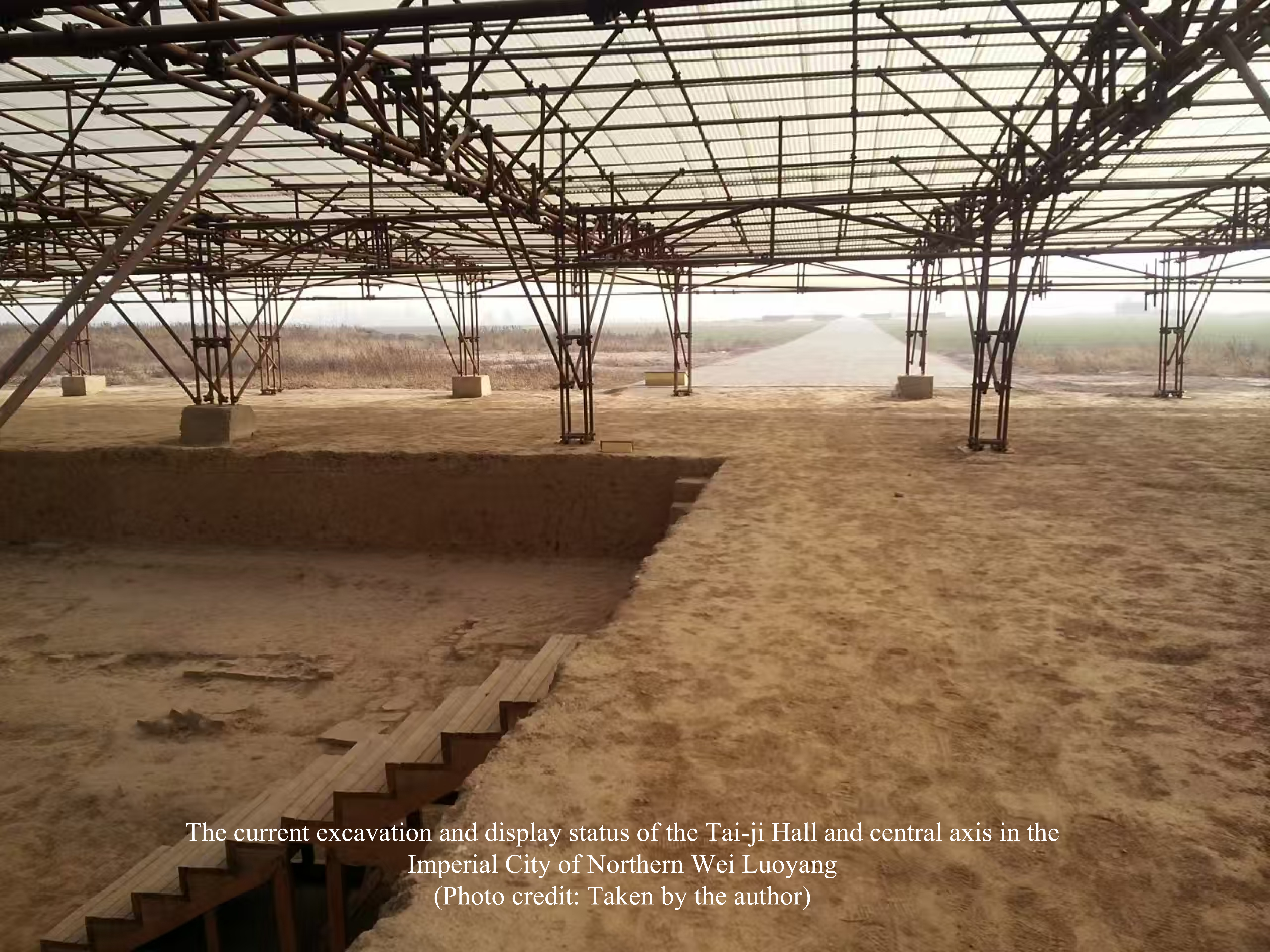

The current excavation and display status of the Tai-ji Hall and central axis in the Imperial City of Northern Wei Luoyang
(Photo credit: Taken by the author)
