# Supplementary material for: Spatial layout characteristics of Northern Wei Luoyang: A spatial humanities analysis of the “Record of the Monasteries of Luoyang”
Source: PLoS One. 2025 May 9;20(5):e0320159. doi: 10.1371/journal.pone.0320159 (PMC12064038; doi:10.1371/journal.pone.0320159)
Supplement: Attachment — Submitted filename: Response to Reviewers.docx [file pone.0320159.s009.docx]

Response to Academic Editor

Dear Dr. Zhang,

Thank you for giving us the opportunity to revise and resubmit our manuscript. We greatly appreciate the insightful comments and constructive feedback provided by you and the reviewers, which have been invaluable in helping us strengthen the quality of our work.

We have carefully addressed each of the points raised, and our responses are detailed in the attached “Response to Reviewers” document. We have also included both a marked-up version of our manuscript, highlighting all revisions, and a clean, unmarked version without tracked changes. These updated documents reflect our commitment to ensuring that our manuscript meets the standards and expectations of PLOS ONE.

Should you have any questions or require additional information, please do not hesitate to contact us. We sincerely thank you once again for your time, guidance, and consideration, and we look forward to the opportunity to further improve and clarify our research.

Yours sincerely,

Wenjia Liu, PhD

School of architecture, Zhengzhou University, No. 100 Science Avenue, Zhongyuan Distric

450001 Zhengzhou, China

+86 13523007270

Response to Reviewer #1

Dear Reviewer #1,

Thank you for your thorough review and thoughtful comments on our manuscript. Your feedback has greatly helped us improve both the quality of our work and our own understanding of spatial humanities research. Below is a summary of how we addressed each of your suggestions:

**1.Expanded critical discussion**

We have revised the Discussion section to include a more in-depth analysis of the three observed spatial morphological features, comparing our findings to other historical Chinese cities. By discussing alternative interpretations and potential explanations for the patterns, we believe our argumentation is now more nuanced and comprehensive.

**2.Addressed limitations and data biases**

In response to your recommendation, we added a new sub-section titled Limitations of Data Sources in the revised Discussion section. There, we provide a candid assessment of the inherent biases and potential shortcomings in the data, clarifying how these issues might affect the study’s outcomes and conclusions.

**3.Elaborated on “why” and “how”**

We have expanded our interpretation of the underlying reasons behind the three morphological features by incorporating historical, socio-political, and cultural contexts. This additional discussion explains the broader significance of these spatial patterns and how they may have emerged over time, providing more insight into the “why” and “how” aspects of our results.

**4.Strengthened the conclusion**

We have revised the Conclusion to offer a more comprehensive overview of our findings, highlighting key contributions and suggesting avenues for future research. We also discuss potential enhancements to data collection and analytical methods that could further refine our approach moving forward.

We sincerely appreciate your meticulous review and believe that your feedback has substantially strengthened our manuscript. Thank you once again for dedicating your time and expertise to improve our work.

Yours sincerely,

Wenjia Liu, PhD

School of architecture, Zhengzhou University, No. 100 Science Avenue, Zhongyuan Distric

450001 Zhengzhou, China

+86 13523007270

Response to Reviewer #2

Dear Reviewer #2,

Thank you for your thorough review and detailed comments on our manuscript. We are truly grateful for the time and effort you spent providing such valuable feedback. Your suggestions have greatly improved the quality of our paper.

Below is a summary of how we have addressed each of your points:

**1.Clarification on the “new digital framework”**

We acknowledge that our original claim regarding a “new digital framework” may have been overstated. In the revised version, we have removed such references and clarified that, while our application may offer fresh insights into Northern Wei Luoyang and other historical Chinese cities, the core methods (GIS, network analysis) themselves are well-established rather than wholly novel.

**2.Enhancements to the Introduction and Literature Review**

We have optimized the Introduction section by including more recent literature relevant to the spatial humanities. We also expanded our discussion of the historical significance of Northern Wei Luoyang, thereby placing our study in a broader scholarly context.

**3.Data Availability**

In the revised manuscript, we provide detailed information on the data we used in GIS and Complex Network analyses, including tables, shapefiles, and Gephi data. We have also deposited these materials in protocols.io for public access, which can be found at:

<https://www.protocols.io/blind/D9EBB57CD04511EFA3430A58A9FEAC02>

The data has not been officially published yet, but a DOI will be provided after it is finalized.

**4.Spatial Analysis Parameters**

At your recommendation, we have added detailed parameters for kernel density analysis, mean centers, and standard deviation ellipses. These details appear in the Supporting Information section(S_5 File) and should provide sufficient information for replicating our analyses.

**5.Software and Licensing**

We confirm that ArcGIS is not freely available, but MARKUS and Gephi are open-source and free to use.

**6.Minor Edits and Typographical Corrections**

We greatly appreciate your careful reading and have made the necessary corrections based on your suggestions.

We hope these revisions meet your expectations and further enhance the clarity and rigor of our work. Should you have any additional comments or recommendations, we would be most appreciative to receive them.

Yours sincerely,

Wenjia Liu, PhD

School of architecture, Zhengzhou University, No. 100 Science Avenue, Zhongyuan Distric

450001 Zhengzhou, China

+86 13523007270
